# The Effectiveness Mechanisms of Carbon Nanotubes (CNTs) as Reinforcements for Magnesium-Based Composites for Biomedical Applications: A Review

**DOI:** 10.3390/nano14090756

**Published:** 2024-04-25

**Authors:** Abbas Saberi, Madalina Simona Baltatu, Petrica Vizureanu

**Affiliations:** 1 Department of Material Engineering, South Tehran Branch, Islamic Azad University, Tehran 1777613651, Iran; 2 Department of Technologies and Equipments for Materials Processing, Faculty of Materials Science and Engineering, Gheorghe Asachi Technical University of Iaşi, Blvd. Mangeron, No. 51, 700050 Iaşi, Romania; cercel.msimona@yahoo.com

**Keywords:** Mg-based composites, carbon nanotube reinforcement, mechanical properties, corrosion behavior, biocompatibility

## Abstract

As a smart implant, magnesium (Mg) is highly biocompatible and non-toxic. In addition, the elastic modulus of Mg relative to other biodegradable metals (iron and zinc) is close to the elastic modulus of natural bone, making Mg an attractive alternative to hard tissues. However, high corrosion rates and low strength under load relative to bone are some challenges for the widespread use of Mg in orthopedics. Composite fabrication has proven to be an excellent way to improve the mechanical performance and corrosion control of Mg. As a result, their composites emerge as an innovative biodegradable material. Carbon nanotubes (CNTs) have superb properties like low density, high tensile strength, high strength-to-volume ratio, high thermal conductivity, and relatively good antibacterial properties. Therefore, using CNTs as reinforcements for the Mg matrix has been proposed as an essential option. However, the lack of understanding of the mechanisms of effectiveness in mechanical, corrosion, antibacterial, and cellular fields through the presence of CNTs as Mg matrix reinforcements is a challenge for their application. This review focuses on recent findings on Mg/CNT composites fabricated for biological applications. The literature mentions effective mechanisms for mechanical, corrosion, antimicrobial, and cellular domains with the presence of CNTs as reinforcements for Mg-based nanobiocomposites.

## 1. Introduction

The urbanization of human societies and lifestyle changes lead to an increase in accidents and unexpected events, and on the other hand, unhealthy diets and the phenomenon of population aging in advanced societies increase the risk of osteoporosis; therefore, the rate of bone fractures is increasing in communities [1,2]. Metals have long been used as implant materials in the medical field [3,4]. The attractiveness of using metals is attributed to their inherent properties, including mechanical strength, toughness, higher wear resistance compared to polymers, and higher formability and elongation than ceramics [5,6]. Traditional metals used in implants, such as cobalt–chromium, titanium, and stainless steel, with properties such as excellent corrosion resistance and a relatively high modulus of elasticity, are known as bioinert materials [7,8,9,10,11,12,13]. However, due to the lack of interaction with the host tissue and the high modulus compared with human bone, such materials have resulted in exacerbated stress shielding and the loosening of the implant [14,15]. Additionally, requiring another surgery to remove the replacement implant causes a delay in the recovery process, psychological stress, and additional costs for the patient [16]. New-generation implants are biodegradable metals based on alloys of Fe, Mg, and Zn that are slowly absorbed by the body or eliminated through the body’s metabolism. Bioabsorbable metals are expected to degrade via biological pathways without the host experiencing adverse reactions from the degradation products [3]. Therefore, bioabsorbable metals focus on how the host metabolizes or absorbs the degradation products. A summary of some of the physical and mechanical properties of existing metallic biomaterials, besides the characteristics of natural bone tissues, is given in Table 1.

**Figure 1:**
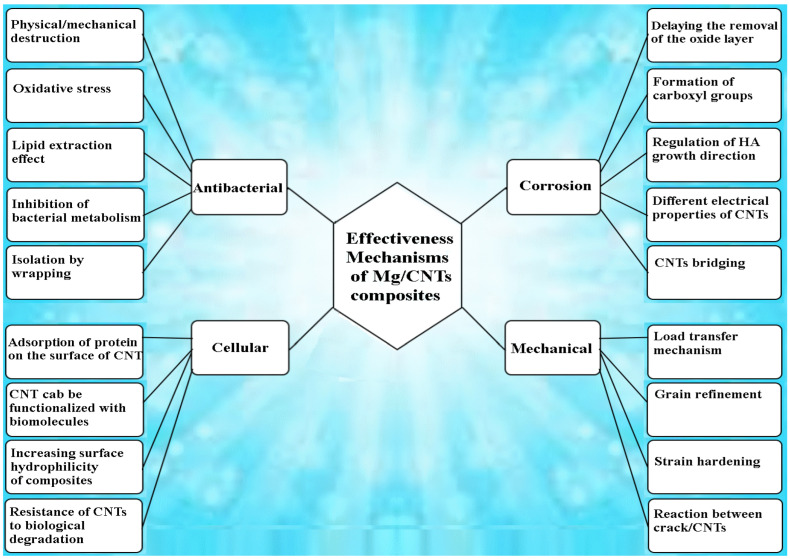
Schematic of effective mechanisms of CNTs on Mg/CNT composite.

Among all biocompatible metals, Mg has the closest elastic modulus to that of natural bone [5]. Mg ranks as the fourth most plentiful cation in the human body, is an essential element for metabolism, and is primarily stored in bone tissue [6]. Stimulating the growth of bone cells and speeding up the recovery of bone tissue is accelerated with a diet containing Mg. Due to the presence of Cl^−^ in the physiological environment of the body, the Mg alloy decomposes, and there is no need for secondary surgery to remove the implant. The corrosion product of Mg implants is Mg^2+^, which does not cause unwanted side effects because excess Mg cations are easily excreted in the urine [25,26,27]. Despite all these advantages, researchers still face significant challenges in their widespread application in orthopedics. For example, Mg alloys must have good mechanical strength when used as implants under load, but in practice, this is not the case: Mg implants degrade in physiological environments with higher rates of bone repair, which is associated with reduced mechanical performance [28,29]. On the other hand, the formation of bacterial biofilms due to the adhesion and colonization of bacteria is known as the peri-implantitis causative factor, leading to implant resorption. However, once a bacterial biofilm is formed, it is difficult for the immune system to destroy it. Research has shown that Mg ions have a relatively strong antibacterial effect against *S. epidermidis* and *E. coli*. The antibacterial effect is mainly caused by Mg ions increasing the osmotic pressure around the bacterial cells [30,31]. However, due to the presence of thick biofilms, bacterial cells can resist external pH changes and cause tissue infection [27,31]. Therefore, due to the complex physiological environment of the implant, changing the pH values alone does not provide an excellent antibacterial effect [15,32]. The new generation of implants includes composites and engineered materials. Composites are made by mixing two or more ingredients, and the resulting mixture has a unique property that no component can have alone. Reinforcements such as metal particles, ceramic particles, and carbon fibers can be used to composite Mg [33,34,35]. In recent years, research on carbon nanofiller materials such as CNTs has attracted attention [36,37,38,39]. CNTs are an attractive option as an additive in composites due to their inherent properties such as Young’s modulus, strength, and very high thermal conductivity [40], in addition to their antimicrobial activity against a wide range of microorganisms [41]. In this review, in each separate section, we examine the mechanical, corrosion, and antibacterial performance and cellular responses of CNTs in Mg-based composites for biomedical applications (Figure 1). Furthermore, the key challenges and future road map are investigated, with attention to research on Mg-based implants.

## 2. Carbon Nanotubes (CNTs)

**Figure 2:**
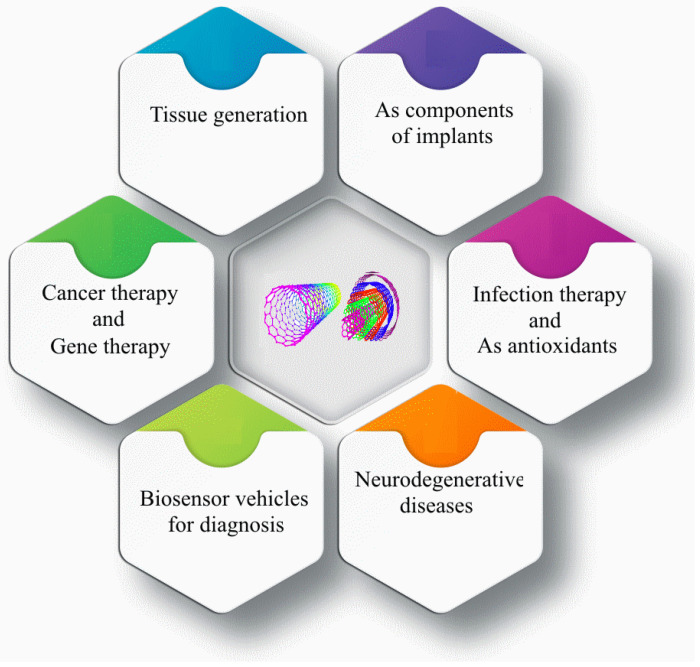
Schematic of carbon nanotubes applications in biomedicine.

Carbon atoms form different allotropes with different bonds and create different types of carbon nanomaterials (CNMs). These include 0D fullerenes, nanodiamonds (NDs), carbon dots (CDs), graphene quantum dots (GQDs), 1D carbon nanotubes (CNTs), 2D graphene and its derivatives, and nitrogen-rich graphene: graphite carbon nitrate (g-CN). CNTs, typical examples of 1D CNMs, are coaxial tubes formed from sp^2^ carbon atoms with a small number of sp3 carbon atoms as defects and can be considered as rolled 1D nanocylinders from 2D graphene sheets. A single graphitic layer or multiple coaxial layers can be rolled up, respectively, into single-walled carbon nanotubes (SWCNTs) or multi-walled carbon nanotubes (MWCNTs) [40]. Carbon monoxide disproportionation and chemical vapor deposition (CVD) are the two most commonly adopted synthesis methods to obtain pristine SWCNTs and MWCNTs, respectively. CNTs are a one-dimensional nanomaterial, with a large specific surface area from 50 to 1315 m^2^/g. The radial dimension of CNTs is nanometer-sized, but the axial dimension is micrometer-sized. CNTs have an aspect ratio of 100 to 1000, much higher than traditional fiber materials, and a low density of about 1.3 g/cm^3^. The covalent bond between the carbon atoms in the ring is the most stable chemical bond that has ever been found in nature, and it has a thermal conductivity of around 3000 W/m/K—a level similar to the thermal conductivity of a diamond. In addition to the electrical and thermal properties of CNTs, their excellent mechanical properties and low density make them highly suitable for use as reinforcing materials to fabricate strong nanocomposites. The researchers showed that CNTs have an elastic modulus comparable to that of diamond, are 100 times stronger than steel, and have a density of just one-sixth that of steel. The outstanding mechanical properties of CNTs stem from the sp2 hybridization of the C-C bond. The elastic modulus of CNTs is approximately 1 TPa—the highest among carbon allotropes. Additionally, CNTs exhibit excellent bendability and plasticity due to their hollow and closed topology [40,42,43,44]. Figure 2 summarizes the main applications of CNTs in pharmaceutical sciences, medicine, biomedicine, and medical engineering [44,45,46].

## 3. Mg/CNT Biocomposites

Mg’s HCP crystal structure makes it less ductile and stronger at room temperature than other low-density metals such as AL and Ti, hindering its wide range of applications [47,48,49,50,51,52,53]. Adding small amounts of nanoscale reinforcement to matrix alloys could be an attractive and practical option for fabricating metal matrix composites with high strength and other advanced properties [54,55,56]. It has been discovered that the unique properties of CNTs, such as high mechanical strength and high thermal and electrical conductivity, have made them an attractive nanofiller for use as a reinforcing material [57,58,59]. The combination of metallic materials (pure or alloy) and carbon nanotubes helps to create nanocomposites by meeting the diversity of functional properties within the same composite. Among the most practical methods of making composites are: (1) powder metallurgy, (2) semi-powder metallurgy, (3) casting with stirring or ultrasonic mixing, (4) thermal spraying, (5) friction stir processing, (6) rolling process, (7) gemini dispersant, (8) electrochemical techniques, etc. [59]. The primary goal of choosing the techniques used should be to solve the main challenges of nanocomposite production, including the uniform distribution of nanofillers and the connection of the effective reaction zone between the nano reinforcer and the matrix. This reaction zone should not be so large that it constitutes the third component of the composite. Each method has advantages and limitations. It seems that the semi-powder metallurgy method has attracted the attention of researchers for the production of Mg-based biocomposites with nano carbon reinforcements due to the possibility of uniform distribution, the opportunity of monitoring during manufacturing, and minor physical damage to the reinforcement [15,60,61,62,63].

## 4. Strengthening Mechanisms and Mechanical Properties

### 4.1. Strengthening Mechanisms of Mg Matrix Alloys

The addition of alloying elements can have a serious impact on the mechanical behavior of Mg. Creating a solid solution, which is a type of alloying, is an effective method to increase the strength of metals. This method increases strength by adding atoms of one alloy element to the crystal structure of another element (base metal), forming a solid solution. Phase and thermodynamic diagrams can provide information about elemental dissolution, phase balance, conversion, and information essential for material design [64,65,66]. Hume-Rothery et al. [66] proposed solid solubility laws for binary alloys based on experimental data on the solid solubility of copper and silver alloys. The proximity of atomic size, electrochemical properties, and crystal structure cause high solubility and otherwise low solid solubility in each other’s matrix phases [66]. The maximum solubility of zinc in Mg is about 2 wt.% at room temperature at equilibrium, and when not more than 2 wt.%, the microstructure is composed of α-Mg. When the zinc content exceeds the solubility limit, the microstructure changes, and many secondary phases are precipitated. With increasing zinc content, a eutectic layer appears in the microstructure of the casting. The very coarse and mostly eutectic structure is distributed mainly in the grain boundaries and less in the inter-dendrite regions [67]. Several studies have been conducted on the effect of zinc additions on the corrosion behavior of Mg. Results show that significant improvements in corrosion resistance are usually achieved by increasing the zinc content to its maximum solid solubility [64,68,69,70]. Calcium (Ca) is another widely used alloying element in Mg for biological applications. In the Mg–Ca binary system, the addition of less than 0.35 wt.% of Ca results in the advantages of a solid solution. In contrast, the addition of Ca above its solubility seriously reduces the corrosion resistance. The addition of amounts exceeding the solubility limit induces the formation of a Mg_2_Ca phase at the grain boundaries and serves as an anode for the matrix phase in electrochemical processes [71].

For Mg–Mn alloys, it was found that the corrosion rate decreases with increasing Mn content in the α-Mg phase. The retarded microgalvanic corrosion, which reduces the potential difference, caused by the increase in the Mg-α phase electrode potential after the Mn solution, could be one of the reasons for the increased corrosion resistance. Furthermore, the formation of Mn-containing oxides and the addition of these oxides to the corrosion product layer help to improve the corrosion resistance of the surface layer and, thus the corrosion resistance of the Mg alloy [72].

In the Mg–RE alloy, the RE elements are also alloyed into Mg alloys to improve corrosion resistance in addition to strength and ductility [73,74]. Since the standard electrode potential of the RE element is close to that of Mg, alloying with the RE element does not significantly change the electrode potential of Mg. In addition, RE elements are known to have an affinity to induce oxidation and, on the other hand, the low chemical activity of surface oxides on Mg alloys. Oxide layers in the vicinity of chlorine-containing solutions can act as a passivation layer to protect the substrate, and after the outer Mg(OH)_2_ layer dissolves, the oxide film also exerts a relatively strong protective effect [74,75].

On the other hand, adding an alloy beyond the solubility limit can lead to the formation of a secondary phase, in which case the strengthening is accomplished by other methods. Precipitation hardening is the phenomenon in which heat treatment at relatively low temperatures increases the hardness of an alloy, resulting in the precipitation of supersaturated solid solution phases or constituents. During this heat treatment process, hard precipitates are finely and uniformly distributed in the soft phases of the matrix, leading to the increased strength of the alloy. The most important requirements for carrying out the age-hardening process on alloys are the limited solubility of precipitates or hard secondary phases in the alloy matrix and the decrease in this solid solubility with decreasing temperature [76,77,78]. The precipitation hardening process depends on the temperature-induced change in solid solubility and produces impurity phase particles that inhibit the movement of dislocations and defects in the crystal lattice, contributing to the hardening of the material [52,79]. These deposits serve the same role as reinforcement in composites [80]. Various processes such as hot extrusion, equal channel angular pressing, and hot rolling are used to improve the mechanical properties of the alloy. Grain size is known to have a significant influence on the mechanical properties of Mg alloys due to their high Hall–Petch reinforcement coefficient (approximately MPa μm ^1/2^) [81,82,83].

### 4.2. Strengthening Mechanisms of Mg/CNT Biocomposites

To date, considerable research has been conducted to evaluate the mechanical properties of Mg/CNT composites. In almost all studies, the most important prerequisites for improving mechanical properties were uniform distribution and good interfacial bonding between the reinforcement and the matrix. Table 1 shows the mechanical properties of the Mg/CNT composites fabricated for orthopedic applications. The presence of CNTs simultaneously activates multiple strengthening mechanisms on the Mg matrix, resulting in improved mechanical performance compared to non-reinforced implants under the same conditions. In this section, each of the identified mechanisms and their effects on Mg/CNT composites are described separately.

**Load transfer mechanism**: The load transfer from the matrix to the reinforcement is generally the most commonly mentioned mechanism in the analysis of strengthening in metal composites. To transfer the load from the matrix to the reinforcement, the elastic modulus of the reinforcement must be higher than the matrix (Er > 2 Em). The load transfer depends on the bond strength between the reinforcement and the matrix as well as the volume ratio and aspect ratio of the CNTs. The CNTs/matrix reaction zone is an important factor in the reinforcement of nanocomposite materials. It is not suitable if there is no reaction between the second phase (reinforcement) and the matrix; in this case, the connection between the second phase and the matrix will be mechanical and weak. On the other hand, if the reaction zone is a large area, this is undesirable because new phases are formed in the composite material, imparting undesirable properties to the desired system [37,84,85].

**Figure 3:**
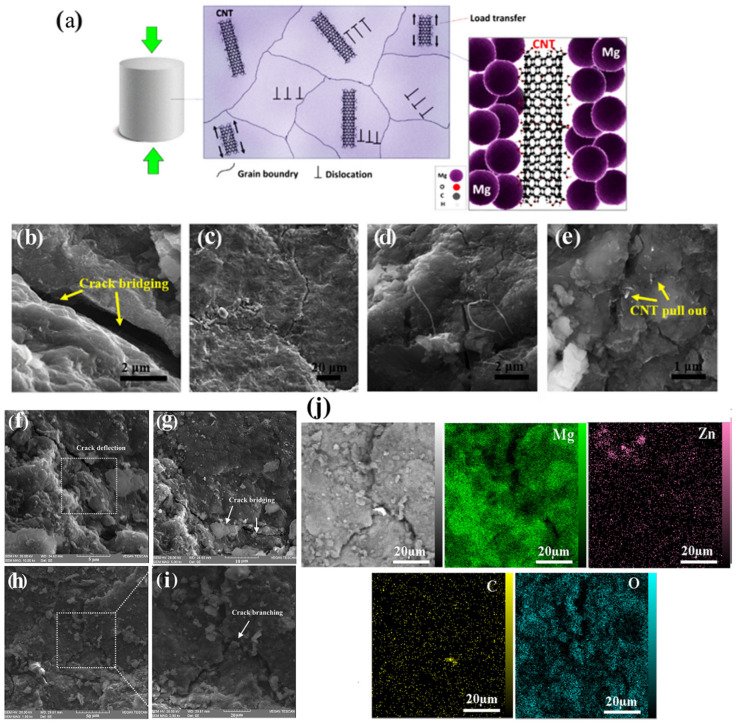
(**a**) Schematic diagram showing the dislocations pinned by CNTs, FE-SEM images of Mg-3Zn/fCNTs (**b**) crack bridging, (**c**) crack branching, (**d**) crack deflection and (**e**) fCNTs pull out [95]. (**f**) Crack deflection; (**g**) Crack bridging and (**h**,**i**) Crack branching mechanisms of Mg/CNT-GNPs composites; (**j**) EDS mapping of Crack branching mechanism [55].

**Grain refinement or texture hardening:** The presence of CNTs as reinforcements causes the grain to be refined and thus improves the mechanical strength. Due to the difference in the melting points of the CNTs and the Mg matrix, they can help reduce the grain size as a nucleator, or at the grain boundary, they significantly affect boundary pinning during manufacturing, resulting in smaller grain sizes in nanocomposites. The relationship between the reduction in grain size and the increase in mechanical strength has been identified as the Hall–Petch effect. This relationship was established on the basis of observations that grain boundaries prevent the displacement of dislocations, and the number of dislocations within the grain also affects stress generation (Figure 3a). Therefore, by varying the grain size, it is possible to affect the accumulation of dislocations within the grain and thus the yield strength [86,87,88]. When the Mg matrix with CNTs as reinforcement is placed at different temperatures during composite fabrication or after fabrication processes such as heat treatment, extrusion, precipitation hardening, etc., CNTs, when pinning and rotating, will affect the movement of grain boundaries and lead to the generation of final microstructures with different grain sizes and/or orientations. The Zener pinning Equation (1) can estimate the mean grain size (D) of the composites [89]:

(1) $$\;\;\;\;D=\frac{kr}{f^{n}}\;\;\;\;\;\;\;\;\;\;\;\;\;$$

where k is a proportional dimensionless constant, f is the volume fraction of the reinforcement, n is the exponent for f in the general form of the Zener equation, and r is the mean reinforcement radius [89]. The CNTs act as a frictional force against the grain boundary migration and hinder grain growth. Grain refinement depends on the CNTs’ diameters, quantity, and length. The stronger pinning effect and then the smaller size of the composite grains are directly related to the reduction in diameter, increase in content, and shortening the length of CNTs [89].

**Strain hardening:** In general, the distribution of the second-phase particles dispersed in the metal can interact with the moving dislocations and increase the strength of the material. Second-phase particles can retard dislocation motion in two distinct ways. If the second phase is small or soft, the dislocations will sever the particles and deform them. The Orowan mechanism can be used to explain the increase in strain hardening when there is the presence of CNTs or non-coherent deposits in the metal matrix. In this case, the dislocations bypass the particles and pass through them by bending the dislocation line. Due to the formation of dislocation rings around the grains, an accumulation of dislocations occurs, and for this reason, in a precipitate-hardened crystal, the strain hardening increases suddenly in the early stages of deformation. Most theories of part strength in the second phase are based on the spherical particles, while the shape of the particles can be important. In an equal volume fraction, the second phase deposit in the form of rods and plates provides twice the strength of spherical particles [90,91,92].

**Reaction between cracks and CNTs:** The best approach to evaluate the mechanism between CNTs and cracks in nanocomposites is in situ tensile and compressive testing. Some of the most common traceable crack/CNT reactions in Mg/CNT composites include CNT bridging (Figure 3b,g), crack branching (Figure 3c,h–j), crack deflection (Figure 3d,f), and CNT pull-out (Figure 3e). During the fracture of the composite, a large number of CNTs are separated from the Mg matrix. On the other hand, CNT pullout suppresses the stress at the crack tip, which can slow crack propagation. CNT bridging applies compressive stress on the crack surface to prevent crack propagation by neutralizing the crack stress, thereby preventing further crack growth and increasing the toughness of the composite. Another useful mechanism that can occur between the CNTs and the crack is “cracking deflection”. When a crack propagates and hits the CNTs, the path is blocked and then the crack path is deflected. Toughness increases by creating a complex path of stress release [41,93,94,95].

It should be noted that the available literature indicates an optimum content of CNTs where the properties of the composite show the greatest improvement, while above this, the deterioration in mechanical properties occurs due to the aggregation of composites [96,97,98]. A number of the mechanical characteristics of Mg/CNTs composites are compared in Table 2.

## 5. Degradation Behavior of Mg/CNT Biocomposites

The amount of Mg ions absorbed by the average person is about 300–400 mg/day, and excess Mg cations in the body can be excreted in the urine [101]. One of the negative effects of Mg alloy degradation is the release of hydrogen into the body. Hydrogen bubbles released by Mg implants cause subcutaneous emphysema [102,103,104]. Subcutaneous emphysema in the body causes a delay in the healing of the injured and surgical area as well as the separation and layering of adjacent tissues [101]. On the other hand, the rate of degradation is usually higher than the rate of bone replacement [105,106]. Therefore, one strategy to solve this problem is to slow down the rate of Mg degradation and corrosion. Although the mechanical properties of CNT-reinforced Mg composites are superior to those of their unreinforced matrix, the corrosion properties of this composite material have not yet been studied extensively. Galvanic pairs due to the difference in the potential of the standard electrode of the Mg and CNTs formed lead to an increase in the corrosion rate. On the other hand, it should be noted that the electrical properties of CNTs have been reported to be different compared to other carbon allotropes [40]. Therefore, the effect of CNTs on Mg corrosion may be different from that of other carbon allotropes [58].

Abazari et al. [95] added CNTs as reinforcements to the Mg matrix. Immersion and corrosion test results showed that cauliflower-like corrosive compounds were deposited on the surface. CNTs can modulate the growth direction of hydroxyapatite (HA) crystals, as CNT particles can control both the nucleation and growth of HA crystals and induce cauliflower-like structures with specific orientations. More specifically, with the presence of fCNTs, the conditions are favorable for the creation of negatively charged carboxyl groups that cause the formation of nucleation sites, attracting Ca^2+^ ions, causing their adsorption, ultimately leading to HA formation. Based on Equations (2) to (5), the rate of Mg degradation is also controlled by the volume of hydrogen gas released and the released Mg^2+^ and OH^−^ ions. As a result, Mg(OH)_2_ is generated on the surface of the Mg alloy [95]:

Anodic reaction:

(2) $$\;\;\;\;{Mg}_{\left(s\right)}\to {Mg}_{\;\;\;\left(aq\right)\;}^{2+}+{2e}^{-}\;\;\;\;\;\;$$

Cathodic reaction:

(3) $$\;\;{2H}_{2}O\;\left(I\right)+{2H}_{\left(aq\right)}^{+}+{2OH}^{-}\;\;$$

(4) $$\;\;{2H}^{+}\left(eq\right)+{2e}^{-}\to H_{2}\left(g\right)\;\;\;\;\;\;$$

Formation of corrosion products:

(5) $$\;\;\;\;\;\;{Mg}_{\;\;(eq)}^{2+}+{2OH}^{-}\left(aq\right)\to {Mg\left(OH\right)}_{2}\;\left(solid\right)\;\;\;\;\;$$

On the other hand, the OH^−^ originally generated by the decomposition of Mg can react directly in physiological solution according to Equation (6), to give

(6) $${HP}_{4\;\;\;\;\;\;\;(aq)}^{2-}+{OH}_{\;\;\;\;\;\;\;(aq)}^{-}\to {PO}_{4\;\;\;\;\;\;(aq)}^{3-}+H_{2}O_{\left(I\right)}$$

**Figure 4:**
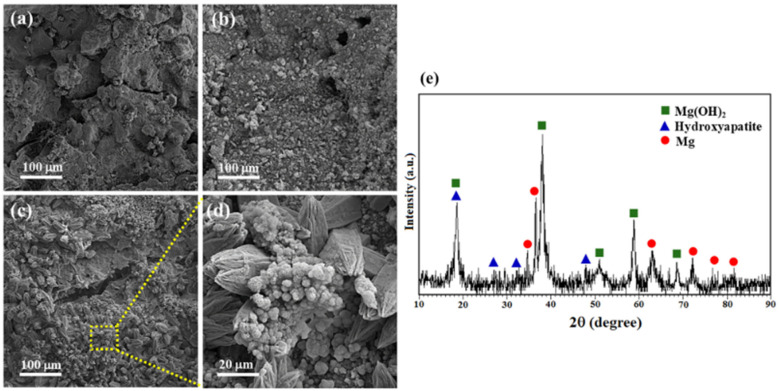
SEM images of (**a**) Mg-3Zn-1Mn alloy, (**b**) Mg-3Zn-1Mn/CNT, (**c**,**d**) Mg-3Zn-1Mn/MgO-CNT nanocomposites after 7 days of immersion in SBF under physiological condition of 5% CO_2_ at 37 °C, and (**e**) XRD pattern of Mg-3Zn-1Mn/MgO-CNTs immersed in SBF for 7 days [99].

Then, ${PO}_{4}^{3-}$ reacts with Ca^2+^ in physiological solution or Mg^2+^ on the composite surface to deposit apatite or Mg-P compounds. The fCNTs are observed to bridge between HA crystals, which can help fill vacancies in the matrix structure, thereby enhancing corrosion resistance [95]. Figure 4 shows the corroded surface morphology of sintered samples of Mg3-Zn-1Mn, Mg3-Zn-1Mn/CNT, and Mg3-Zn-1Mn/MgO-CNT nanocomposites after 7 days of immersion in SBF solution. Corrosion cracks appeared due to pitting corrosion in the Mg3-Zn-1Mn alloy sample (Figure 4a). The Mg alloy forms a thick film of corrosion products, while the Mg-3Zn-1Mn/CNT composite shows microcracks and smaller corrosion spots as well as the existence of needle-like chlorides (Figure 4b). Compared with the Mg3-Zn-1Mn alloy and the Mg3-Zn-1Mn/CNT composite, only a few corrosion spots formed on the Mg3-Zn-1Mn/MgO-CNT composite, indicating that the corrosion potential is much milder, and the corrosion product film is more uniform (Figure 4c). Figure 4e shows the XRD analysis of the Mg3-Zn-1Mn/MgO-CNT composite after 7 days of immersion in SBF at 37 °C [99].

## 6. Antibacterial Performance of Mg/CNT Biocomposites

Recently, many researchers have been looking for alternative antibacterial agents to overcome the challenge of bacterial resistance to existing antibiotics. Among other things, attention has been drawn to nanomaterials as antibacterial agents and their significant potential in controlling infectious diseases. Recently, carbon-based nanomaterials (CNMs) have garnered interest because of their distinct characteristics and comparatively greater biosafety than other antibacterial nanomaterials. Physical/mechanical damage, oxidative stress, lipid extraction, inhibition of bacterial metabolism, and isolation by wrapping when combining CNMs with other materials are among the most important mechanisms of antimicrobial activity of these substances.

**Physical/mechanical destruction:** the outer membrane, or bacterial cell wall, is an essential component of the bacterial cell, responsible for maintaining cell shape, osmotic regulation, protection against mechanical stress, and resistance to infection. Physical/mechanical damage to the outer membrane or bacterial cell wall can lead to dysfunction and the leakage of cytoplasmic components, ultimately causing bacteriostatic and bactericidal effects. Carbon-based nanomaterials (CNMs) of varying sizes can induce physical harm to the outer membrane or cell wall of bacteria. Nonetheless, the degree of mechanical impact from CNMs is linked to their different sizes, possibly resulting from variations in the interaction between CNMs and the bacterial cell wall. Moreover, the effect of mechanical damage can be affected by factors like the thickness, length, and dispersion of CNMs, as well as the method of bacterial culturing, like stirring speed. Therefore, CNTs with small diameters have a higher ability to have a needle-like impact on bacterial cell walls than CNTs with large diameters. Compared to a certain weight fraction of single-layer CNTs, shorter CNTs show relatively sharper tips than longer CNTs. Therefore, the risk of mechanical disruption is increased by its sharp tip to induce antibacterial activity. Unlike agglomerated CNTs, well-dispersed CNTs act as “nanoparticles” and can significantly enhance the penetrating effect on bacterial cell membranes. Therefore, CNTs with smaller diameters and lengths, uniform distribution, and higher stirring speed will lead to more effective antibacterial effects through the mechanism of mechanical damage to the cell wall [41,43,55].

**Oxidative stress:** there are two types of processes; ROS-dependent processes and ROS-independent processes. In the first case, CNMs cause ROS-dependent oxidative damage through a photodynamic process. In the absence of light, ROS generation is initiated by electron transfer from biological electron donors (e.g., NADH) to O_2_ through CNMs (e.g., carboxylated SWCNTs). ROS generated in either case can cause the inhibition or killing of bacteria through protein inactivation, lipid peroxidation, and nucleic acid damage [42,107,108,109].

**Lipid extraction effect:** Like mammalian cells, bacterial cells have lipid membranes that are necessary for absorbing nutrients and eliminating toxic molecules. Therefore, disruption of membrane structure and its functional integrity leads to bacterial inhibition or death. In contrast to the direct physical disruption of the bacterial cell wall or outer membrane by the nanodarts/blades/knives described above, CNMs, with a high concentration of surface active sites, can penetrate the lipid membrane of bacteria and extract most of the phospholipids on the bacterial surface [110].

**Inhibition of bacterial metabolism:** Bacterial metabolism involves processes that convert nutrients into energy and remove waste molecules. The life, growth, and reproduction of bacteria are the result of this mechanism. Unlike eukaryotic cells, bacteria do not have mitochondria and therefore the reactions involved in energy metabolism involving the respiratory chain and ATPase are centralized in cell membranes. Conductive CNMs exert antibacterial effects by removing electrons from the membrane respiratory chain [110].

**Figure 5:**
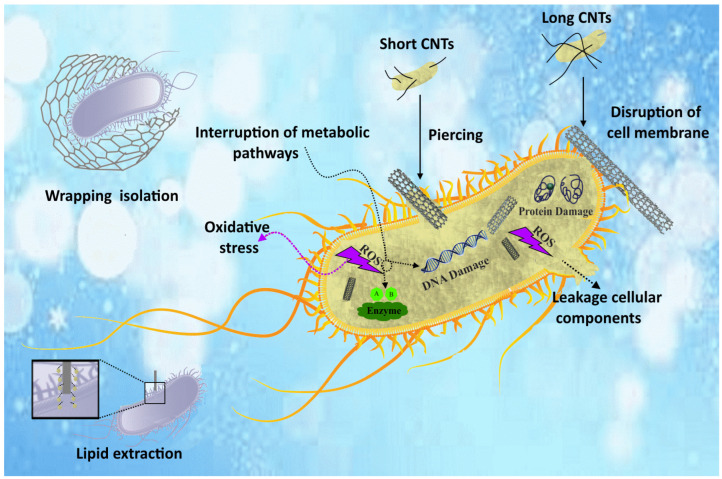
Schematic of the antimicrobial mechanism of CNT reinforcement.

**Isolation by wrapping:** In addition to the antibacterial effect that directly damages bacterial cells, some CNMs with large surface areas can also surround bacterial cells and isolate them from the nutritional environment, thereby leading to bacterial inactivation. Although the wrapping effect was first identified when studying the antibacterial performance of GNPs, it is also a common process in long SWCNT fibers [110]. The schematic of different antimicrobial mechanisms of CNTs is shown in Figure 5.

## 7. Cellular Response of Mg/CNT-Based Composites

Biocompatibility and biodegradability are important requirements for materials capable of temporarily replacing damaged body tissues. Mg is an essential mineral for human metabolism, and its deficiency has been associated with various pathological conditions. The adult human body contains approximately 1 mole (21–28 g) of Mg, and the human body’s daily requirement is approximately 350 mg [111,112]. Therefore, Mg enters the body’s metabolic cycle or is excreted in urine. In recent years, technological advances in the production of engineered materials, especially nanocomposites, have made it possible to create multifunctional properties. However, because the results of studies on their toxicity are inconclusive, from the standpoint of biomedical purposes, the biological properties and interactions of these nanomaterials in biological or simulated environments with the body have left many doubts. In this regard, further research on the biological properties of these composites, including cellular response, biocorrosion, and cytotoxicity, appears to be necessary [113].

The available safety data collectively indicate that CNTs are of low toxicity via various exposure pathways for biomedical applications. CNTs induced meaningful toxicity only when a very high dosage (60 mg/kg) under the Polyethylene-Glycol-MWCNT (PEG-MWCNTs) form was administered. On the other hand, when CNTs were used as tissue engineering materials for cell growth by implanting subcutaneously, CNTs exhibited very good biocompatibility and did not cause any serious toxicity [114]. In fact, there are a few parameters influencing the toxicity of CNTs in vivo [45]. Metal impurities might contribute partially to oxidative stress, and thus, the careful purification of CNTs is necessary. Chemically functionalized CNTs show higher biocompatibility than pristine CNTs. Thus, Yang et al. [115] reported that functionalized CNTs are generally biocompatible and have low toxicity for biomedical applications. Further cytotoxicity evaluation tests are encouraged to determine the safety threshold value of different CNTs and clarify the toxicological mechanism [45].

CNTs can play an important role as components in the production of artificial tissues and implants because compared to other existing materials, CNTs are resistant to biological degradation and can be functionalized with biomolecules for enhancing organ regeneration [45]. In tissue engineering, applications of CNTs and graphene have been explored for central nervous system regenerative interventions and orthopedic implants. The interaction between proteins and CNTs depends on several factors such as electrostatic attraction, hydrogen bonding, and the hydrophobicity or wettability of CNTs. The adsorption of proteins on CNTs is also dependent on the type of protein, the surface charge of the protein and CNTs, as well as the pH, ionic strength, and temperature of the surrounding environment. Moreover, π-π stacking caused by strong van der Waals forces has been recognized as an important adsorption mechanism of proteins on CNT surfaces, and π-π stacking typically occurs between the strong sp^2^ bonding of carbon atoms in CNMs and the benzene rings in amino acids (building blocks of proteins). One of these proteins is bone morphogenetic protein (BMP), which is known to increase the bone differentiation of metal implants and has excellent adsorption capacity on CNT surfaces.

The biosafety of these particles is ensured by the immobilization of CNTs in the metallic biomaterial, which prevents them from being deposited directly into the surrounding tissue. Further and more comprehensive research appears to be necessary for their safe clinical use as components of novel biomaterials for orthopedic applications, as CNTs are safe when applied topically but not in specific areas, such as the lungs and abdominal cavity [93]. Given the importance of the surface area in the discussion of toxicity and compatibility of carbon nanotubes in solvents, the surface quality of nanotubes may have a favorable impact on their dispersion in matrices as well as their compatibility. Several research groups have considered carbon nanotubes a very suitable option for use in simulation and clinical treatments of nerve injury. This is due to their negative charge and ability to reconnect nerve cells after injury [116,117,118].

**Figure 6:**
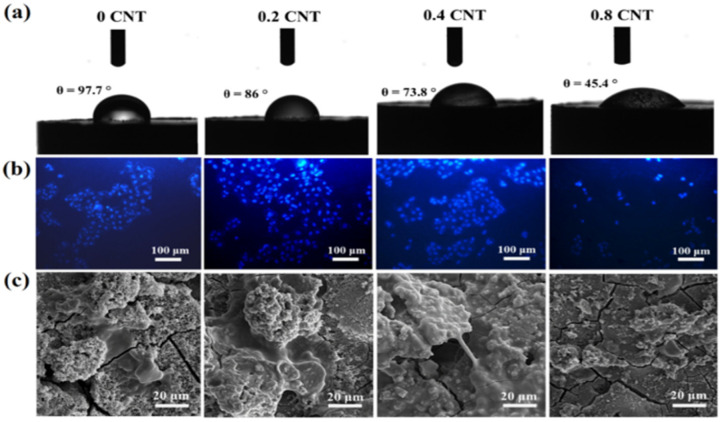
(**a**) Images of water contact angle, (**b**) Fluorescent DAPI staining ofMG-63 cells grown after 24 h, and (**c**) SEM images of the morphology and adhesion of these cells for 3 days on Mg–3Zn alloy matrix, Mg-3Zn/0.2fCNTs, Mg-3Zn/0.4fCNTs and Mg-3Zn/0.8fCNTs nanocomposites [95].

It has been found that composites containing CNTs can lead to the hydrophilicity of the composite surface by forming functional groups such as carboxyl, carbonyl, and hydroxyl. This phenomenon is directly related to the improvement of biocompatibility because by improving the hydrophilicity of Mg-based composites, biocompatibility increases. This may significantly contribute to medical success, as increasing the wettability of the implantable composite surface improves its ability to adhere to biological materials. Increasing the hydrophilicity of the bone implant surface is beneficial for the absorption of nutrients and bioactive factors, thereby improving bone repair during in vivo implantation [41,119,120]. By adding fCNTs to the Mg-3Zn matrix, Abazari et al. [95] studied their biological behavior for use in load-bearing implants. According to Figure 6a, the hydrophilic property is improved by adding CNTs. The formation of a large number of oxygenated functional groups shows the hydrophilic nature of carbon materials. Composites with high CNT content can form large agglomerates, leading to a higher degree of hydrophilicity and causing galvanic attack and surface cracking. Therefore, corrosion resistance decreases with increasing hydrophilicity. The cells in 0.2 and 0.4 fCNT extracts did not have significant differences in density and morphology; therefore, it can be said that fCNTs did not have any harmful effects on density and morphology. According to Figure 6b, it was observed that the addition of 0.8 wt% fCNTs has the opposite effect in increasing the cell nucleus and in other words reducing cell viability. Figure 6c shows osteoblastic cells (MG-63) on different samples. The expanded and flattened morphology of cells in composites up to 0.4 wt% fCNTs showed non-toxicity and good cell compatibility. In the Mg-3Zn/0.8fCNT composite, the osteoblasts were reduced and had a relatively rounded morphology (Figure 6c). Thus, it can be concluded that the composites with high fCNT content have a negative effect on the adhesion, spread, and proliferation of osteoblasts.

## 8. Summary and Future Road Maps

This review article aims to present the mechanisms and interactions between carbon nanotubes as reinforcements and biodegradable Mg as a matrix. Carbon nanotubes have special properties such as high specific surface area, very high diameter/length ratio, very high Young’s modulus, strength, and thermal and electrical conductivity. The above properties have led researchers to study and explore carbon nanotubes for about two decades as reinforcements for metal-based composites for industrial use. However, according to recent research on the biological mechanisms of CNMs, it seems that the use of CNTs as an effective element to solve the challenges of biocompatible metals is feasible. According to the available literature, limited research has been conducted on biodegradable Mg composites containing CNTs as reinforcing materials for orthopedic applications. Most research has focused on manufacturing methods such as casting and powder metallurgy to develop biodegradable Mg matrix composites. Some studies focus more on the mechanical properties and strengthening mechanisms, while others focus more on the corrosion and biological behavior of Mg/CNT-based composites. Research results show that the mechanical properties of composite materials are significantly improved when adding CNTs. The CNTs/matrix contact surfaces can be affected by mechanisms such as the efficiency of charge transfer from the Mg matrix to the CNTs core during mechanical deformation, grain refinement or texture hardening, strain hardening, and reaction between cracks and CNTs. The corrosion properties of this composite material have not yet been studied in depth. The corrosion rate increases due to the large difference between the potentials of the standard electrodes of Mg and CNT, leading to the formation of galvanic couples between them. However, CNTs can facilitate the growth sites of hydroxyapatite (HA) crystals because CNT particles can induce cauliflower-like structures with specific orientations. Several mechanisms have been mentioned in the literature to explain the antibacterial effect of CNTs, all these come from the unique physicochemical properties of carbon. However, the presence of CNTs as effective substances in medical applications remains controversial and complex. It seems that strong interdisciplinary collaboration can lead to promising developments and results so that CNTs can be more widely used as one of the components in medical devices.

## Figures and Tables

**Table 1:** Comparison of physical and mechanical properties of bone tissues with existing non-degradable and biodegradable metal materials.

Material Type	Quantitative Parameters							Qualitative Parameters		Refs.
	D (g/cm^3^)	E (GPa)	ε (%)	UTS (MPa)	Y.S	UCS (MPa)	D.R (mm y^−1^)	Advantages	Disadvantages	
Cortical human bone	1.8–2.1	3–30	1–4	35–283	70–100	164–200	NBR	-	-	[17,18]
S.S	7.9–8.1	170–205	10–40	460–1700	300–400	500–1000	No	High strength Low cost	Low wear resistance High Young’s modulus MRI artifacts because of magnetism	[17,19]
Ti alloys	4.4–4.5	110–127	10–15	800–1200	700–900	900	No	High strength High corrosion resistance	Low wear resistance Toxic alloying elements, e.g., Al, V	[20,21]
Co–Cr alloys	7.9–9.2	200–240	8–20	700–1300	480–550	450–1000	No	High strength	Toxicity (Co-Cr ions). High Young’s modulus	[2,20]
Mg alloys	1.74	41–45	5–40	135–285	130–250	65–100	0.8–2.7	High biocompatibility. Excellent biodegradability. Good tensile strength Elastic modulus close to bone Density close to bone	High corrosion rate Less compressive strength than bone Release of hydrogen gas during degradation	[17,22,23]
Zn	7.1	78–121	0.3–2	100–150	21–27	30–100	0.1–0.3	Good biocompatibility Good biodegradability Moderate corrosion rate Low melting point	Poor mechanical properties Age hardening	[2,24]
Fe	7.8	213	37.5	300	120–150	560	0.1	Good biocompatibility biodegradability Excellent strength No gas generation during degradation	Very slow corrosion rate Possibility of mass corrosion product causing re-accumulation in the tissue High Young’s modulus	[8,22]

D: density; UCS: ultimate compressive strength; UTS: ultimate tensile strength; E: Young’s modulus; **ε**: elongation; YS: yield strength; D.R: degradation rate; NBR: natural one remodeling.

**Table 2:** The mechanical properties of the Mg/CNTs composites fabricated for orthopedic applications.

Sample	Processing Route	Applications	UCS (MPa)	Hardness (HV)	Years	Ref.
Mg-3Zn	SPM + SPS	Biodegradable implants	122 ± 6	59 ± 2.3	2023	[94]
(Mg-3Zn)94.5/Br5-CNTs0.5	SPM + SPS	Biodegradable implants	185 ± 9	79 ± 3.1	2023	[94]
(Mg-3Zn)89/Br10-CNTs1	SPM + SPS	Biodegradable implants	210 ± 10	93 ± 3.6	2023	[94]
(Mg-3Zn)83.5/Br15-CNTs_1.5_	SPM + SPS	Biodegradable implants	110 ± 5.5	65 ± 2.4	2023	[94]
Mg-6Zn	SPM + SPS	OFFD	156	49.5	2023	[93]
Mg-6Zn/5TiO_2_-0.5MWCNTs	SPM + SPS	OFFD	221	68	2023	[93]
Mg-6Zn/10TiO_2_-1MWCNTs	SPM + SPS	OFFD	269	79	2023	[93]
Mg-6Zn/15TiO_2_-0.5MWCNTs	SPM + SPS	OFFD	233	82	2023	[93]
Mg-6Zn	SPM + SPS	Implants	145 ± 7	50 ± 1.7	2023	[55]
Mg-6Zn/0.25GNPs-0.25CNTs	SPM + SPS	Implants	210 ± 10	68 ± 2.3	2023	[55]
Mg-6Zn/0.5GNPs-0.5CNTs	SPM + SPS	Implants	255 ± 12	76 ± 2.6	2023	[55]
Mg-6Zn/1GNPs-1CNTs	SPM + SPS	Implants	115 ± 6	78 ± 2.7	2023	[55]
Mg-2.5Zn-0.5Zr	SPM	Biomedical devices	151 ± 7.5	58	2022	[41]
Mg-2.5Zn-0.5Zr/0.3CNTs	SPM	Biomedical devices	196 ± 9.8	67	2022	[41]
Mg-2.5Zn-0.5Zr/0.6CNTs	SPM	Biomedical devices	237 ± 12	78	2022	[41]
Mg-2.5Zn-0.5Zr/0.9CNTs	SPM	Biomedical devices	148 ± 7	80	2022	[41]
Mg-3Zn	SPM + HTE	Load-bearing bone implants	289.6 ± 13	66 ± 2	2022	[95]
Mg-3Zn/0.2fCNT	SPM + HTE	Load-bearing bone implants	368.2 ± 12	70 ± 2	2022	[95]
Mg-3Zn/0.4fCNT	SPM + HTE	Load-bearing bone implants	390 ± 15	74 ± 2.5	2022	[95]
Mg-3Zn/0.8fCNT	SPM + HTE	Load-bearing bone implants	320.2 ± 14	76 ± 3	2022	[95]
Mg-3Zn-1Mn	SPM + HTE	Medical implant	295.6 ± 20.4	51.6	2022	[99]
Mg-3Zn-1Mn/CNTs	SPM + HTE	Medical implant	404.8 ± 16.1.	74.5	2022	[99]
Mg-3Zn-1Mn/CNTs-MgO	SPM + HTE	Medical implant	429 ± 15	83.4	2022	[99]
Mg	PM	Biodegradable implants	-	36.9	2019	[100]
Mg/0.1% MWCNTs	PM	Biodegradable implants	-	43.6	2019	[100]
Mg/0.2% MWCNTs	PM	Biodegradable implants	-	43	2019	[100]
Mg/0.3% MWCNTs	PM	Biodegradable implants	-	46.5	2019	[100]
Mg/0.5% MWCNTs	PM	Biodegradable implants	-	37.5	2019	[100]

SPM: semi-powder metallurgy; SPS: spark plasma sintering; HTE: hot extrusion; OFFD: orthopedic fracture fixation devices; PM: powder metallurgy.
